# Model-based learning retrospectively updates model-free values

**DOI:** 10.1038/s41598-022-05567-3

**Published:** 2022-02-11

**Authors:** Max Doody, Maaike M. H. Van Swieten, Sanjay G. Manohar

**Affiliations:** Nuffield Department of Clinical Neurosciences, University of Oxford, Oxford, UK

**Keywords:** Decision, Human behaviour

## Abstract

Reinforcement learning (RL) is widely regarded as divisible into two distinct computational strategies. Model-free learning is a simple RL process in which a value is associated with actions, whereas model-based learning relies on the formation of internal models of the environment to maximise reward. Recently, theoretical and animal work has suggested that such models might be used to train model-free behaviour, reducing the burden of costly forward planning. Here we devised a way to probe this possibility in human behaviour. We adapted a two-stage decision task and found evidence that model-based processes at the time of learning can alter model-free valuation in healthy individuals. We asked people to rate subjective value of an irrelevant feature that was seen at the time a model-based decision would have been made. These irrelevant feature value ratings were updated by rewards, but in a way that accounted for whether the selected action retrospectively *ought* to have been taken. This model-based influence on model-free value ratings was best accounted for by a reward prediction error that was calculated relative to the decision path that would most likely have led to the reward. This effect occurred independently of attention and was not present when participants were not explicitly told about the structure of the environment. These findings suggest that current conceptions of model-based and model-free learning require updating in favour of a more integrated approach. Our task provides an empirical handle for further study of the dialogue between these two learning systems in the future.

## Introduction

Within the mammalian brain it is believed that two learning systems guide behaviour. Model-free learning involves reinforcing actions that lead to reward via a simple temporal difference learning mechanism^1, 2^, whereas model-based learning incorporates a cognitive map^3^ of the world to guide behaviour. While these two systems must interface to produce coherent behaviour, it is highly debated when and where this integration occurs^4^. Despite a historical consensus that these two systems interact only at the time of decision, recent findings now favour the view that they interact during learning^5–7^.

Model-free learning acquires action values based on reward prediction errors (RPEs). Action value, the predicted reward associated with an action, is updated by means of an RPE to improve the accuracy of the value estimate^8^. Model-based values, in contrast, are the values of future states rather than immediate actions, and so are tied to the external structure of the environment and the internal model. A key variable in driving these environmental maps in model-based learning is thought to be a state prediction error, which is produced when there is a conflict between the external structure of the environment and the internal model^9^.

To investigate the neural basis of model-free and model-based learning, Daw et al.^5^ devised a two-stage decision task. In this task, participants make two sequential decisions to obtain a reward. The first is always between the same pair of options, whereas the pair of options offered for the second decision depends on the choice made at the first stage. The transitions between the first and second stage are probabilistic, with one pathway more common than the other. If participants are driven by model-free RL, they will repeat decisions in the first-stage that led to a reward, whereas model-based agents will choose actions that commonly transition to states where rewards have been obtained. Consequently, the use of model-free and model-based approaches produce opposing behaviours that are quantifiable.

Two concepts are traditionally used to think about model-based behaviour in the two-stage decision-task: model-based learning and model-based decision-making. Model-based *learning* refers to two processes: the learning of transitions and the structure of the task through state prediction errors (state learning), and subsequently, learning the value of the stage 2 states (state value learning). These processes consequently result in the production and refinement of an internal model of the task structure. Once the structure of the task is learned, participants only need to update values of stage 2 states, because they prospectively assess stage 1 state values at the time of decision. This value updating does not incorporate the transitions. The second process—model-based *decision-making*—refers to how participants make choices utilising the model. At the time of the stage 1 decision, model-based individuals make use of their internal model prospectively to assess which path is more likely to lead to the stage 2 state with the highest state value. This prospective step is comparatively cognitively demanding, and therefore costly, when compared with the learning of state values after each trial. It is generally assumed that this *forward planning* is the only way individuals may utilise an internal model in the task.

However, this is not the only approach by which apparent model-based behaviour may arise in this task. At the time of reward, subjects could *retrospectively update* the action value of the stage 1 choice depending on whether they received a common or rare transition trial. On the next trial, at the time of decision, the most valuable action would then be selected. In this approach, reward is propagated back through the causal chain to the action that is most likely to lead to a reward, taking into account the transition probabilities. For example, imagine you take an umbrella with you on a summer’s day, and it happens to rain heavily. The retrospective updating approach would predict that you might engage an internal model. The internal model would recognise that rain in summer is a rare event, and, instead of reinforcing carrying an umbrella in summer, it would reinforce the action of taking an umbrella with you on a winter’s day when rain is far more likely.

At the core of such a process would be a type of model-based RPE, that is used to update the value not of the chosen action, but rather of the action that most likely *would have led* to the current state. In the simplest case, this would generate a prediction error at the time of outcome relative to the model-free value of the action that *commonly* leads to the actual stage 2 state. In other words, “backward-planning” could be used to update the stage 1 model-free values when reward is received. More sophisticated accounts may involve mental replay of actions^10–13^ permitting truly “offline” learning. There are clear differences between this type of model-based action value learning and traditional model-based learning and decision-making. In standard model-based learning and decision-making, the learning of state values is cheap, and the computation at the time of decision is costly. In contrast, in the retrospective model-based action-value updating approach, the cost is primarily incurred at the time of reward, when action values are updated retrospectively.

Computational approaches in line with this concept of model-based influence on model-free value estimates have already been well-studied within the DYNA architecture^14^. In DYNA, the model-based system trains the model-free system by replaying experienced state-action pairs offline and simulating the rewards and transitions using an internal model in order to update model-free value estimates. Consistent with this idea, representations in the hippocampus may ‘replay’ sequences of states^13^. Recent work in rodents^15^ and in humans^16^ suggests that replay may act as a substrate for exploring past or future trajectories through the environment. Magnetoencephalogram recordings reveal a backward replay signal 160 ms after reward which was associated with learning the value of choosing an action on a path not taken by the individual^11^. In other words, replay may provide a means of assigning value using the task structure to events even if the participant did not experience them.

Three pieces of behavioural evidence also raise the possibility that a cognitive map in humans may influence model-free value. First^6^, peoples’ preferences can be influenced by changes in future state values, even when first-stage decisions are made without any later-stage choices or rewards, even in situations where an optimal planner should actually ignore future states. This could be interpreted as state values guiding choice, without planning. This is abolished by cognitive load during learning, suggesting a DYNA-like offline replay may be responsible. One mechanism of re-valuation might be that people evaluate a state in terms of the expectancy of future states that might result^17, 18^. Second^12^, people tend to repeat choices that causally led to reward, even if the feature that mediates this causal effect is irrelevant to the next decision. The influence of task-structure on model-free value is required to explain such a finding. Finally, there is evidence that reward is attributed to actions not from memory, but from a model-based simulation of possible histories^19^. Decisions in the standard two-stage decision task fit better to model-free learning where an internal model determines which actions are eligible for reinforcement (retrospective assignment), rather than forward planning. In this framework by Toyama et al., replay may actually occur at the time of reward, and can be captured by an “eligibility adjustment”^19^. Taken together, this evidence suggests that model-based and model-free systems do not operate independently. We opted for a more direct way than these tasks to examine retrospective updates by probing how cached values are attributed to stimuli during the two-stage task. To do this, we included features that were irrelevant to the decision in the stage 1 stimuli, and asked participants to report model-free value estimates of these features. We examined how these subjective values changed over the course of learning. This allowed us to examine whether the cached value estimate is influenced by reward, and in particular, whether the updates might be model-based, i.e. reflecting whether the state encountered was likely or unlikely to follow the option that was selected. A model-free system is by definition blind to this, so such an effect would reflect model-based training of the model-free system.

We also sought to investigate factors affecting this interaction. Model-based performance may depend closely on explicit knowledge and working memory (WM). Accordingly, it requires attentional resources^20, 21^, and is vulnerable to transcranial magnetic stimulation in left dorsolateral PFC^22^. We therefore hypothesised that transfer value from the model-based to the model-free system may be an explicit, attention-dependent phenomenon. If so, we might expect transfer to also correlate with WM capacity, to be attenuated by attentional demands, and to be abolished in the absence of explicit knowledge of task structure.

## Experiment 1: Healthy participants show model-based transfer of value

### Method 1

#### Participants

We recruited a cohort of healthy subjects (n = 30) aged 18–35 to assess the presence of model-based transfer using the Oxford Psychology Research (OPR) participant recruitment scheme. Each participant gave written informed consent. Participants had normal or corrected-to-normal vision and were paid £10.80 for participating. All experimental protocols were approved by the Oxford Medical Sciences Interdivisional Research Ethics Committee of the University of Oxford, reference R45265/RE001. All methods were performed in accordance with the relevant guidelines and regulations.

#### Task design

**Figure 1:**
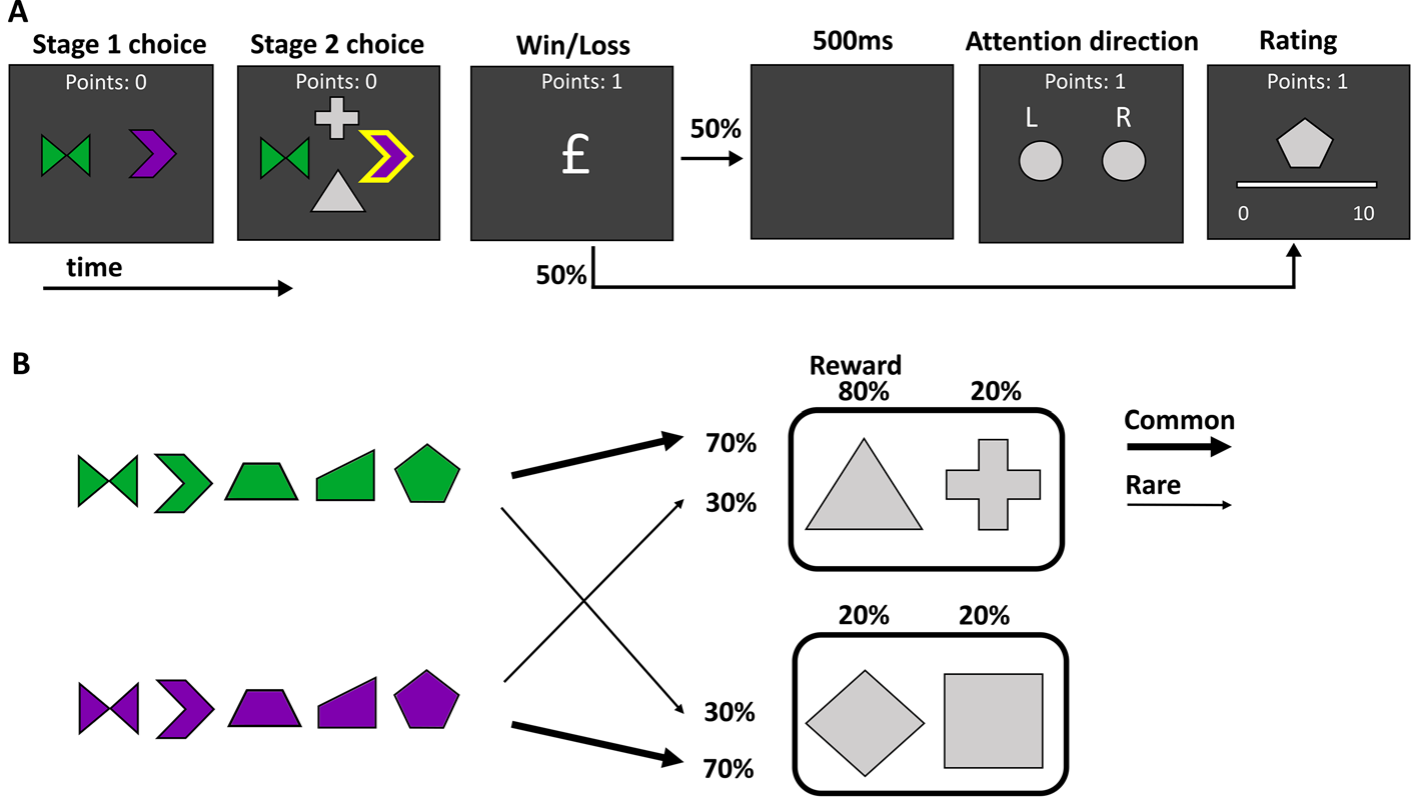
Structure of the task. (**A**) Participants chose one of two coloured shapes, which led to a second decision between one of two pairs of grey shapes. At any particular time one of these grey shapes was most likely to be rewarding. On half of trials participants were asked to remember back to their stage 1 choice to recall if the shape chosen was on the left or right side of the screen. On the other half of trials participants proceeded directly to the shape ratings screen, where participants rated how likely stage 1 shapes were to be rewarding at that time. (**B**) The probabilistic relationship between stage 1 colour and stage 2 shapes. Colour alone determined the transition probabilities to stage 2 pairs. The five stage 1 shapes were randomly presented and were outcome-irrelevant, making them a useful conduit for the passive accumulation of value over time. During a 32-trial block, one of the four grey shapes was most likely to be rewarding, in this case the triangle.

Participants performed an adapted version of the two-stage decision task^11^, which allows for the dissociation of model-free and model-based components in decision-making^1, 23–28^. All experiments were performed using a touchscreen PC running MATLAB R2018b. Subjects made two sequential choices (Fig. 1A). In the first stage, a choice was made between two coloured shapes, leading to one of two second-stage states. Each *colour* leads more frequently (70%) to one of the second-stage states (a common transition), and, in a minority of the choices (30%), to the other second-stage state (a rare transition) (Fig. 1B). The stage 1 shapes are not relevant to the transitions to stage 2 states. The second-stage states require a choice between two grey shapes. Only one of the second-stage shapes was more likely to lead to a reward, which changed randomly every 32 trials. This was implemented to dissociate model-free and model-based learning strategies^29, 30^. The probability of reward for the most rewarding grey shape was 0.8, whereas other shapes had a 0.2 chance of producing a reward. After each second-stage choice, participants received feedback about the outcome of the choice. The total accumulated points were continuously displayed at the top of the screen. The location and identity of the first and second-stage stimuli varied across trials and were counterbalanced for each participant. A time limit of 4 s was applied to the first choice but did not apply to other stages. If participants failed to enter a choice within the time limit, the trial was aborted and not included in further analyses. The stage 1 shapes were never repeated on the next trial. The full task consisted of 256 trials.

Two new features were added to the standard task. First, we introduced five shapes to the first-stage choice and assessed whether these stage 1 shapes, which do not have an inherent value, acquire a value throughout the task. Participants were explicitly instructed that “*the colour of the stimulus, rather than its shape, determines the probability of reaching the options in the second choice*”. At the end of a trial, participants were presented with one of the five stage 1 shapes in grey and asked to “*assign a value between 1 and 10 to indicate the likelihood the presented shape was rewarding at that time*” on a visual analogue scale. Crucially, the shape of the first stage stimulus had no predictive effect of reward, meaning that these shapes should not acquire any value in principle. By probing the subjective value trial-by-trial, we were able to track changes in value throughout the task^31^. Participants were not asked to rate shapes on the first 10 trials.

Second, we manipulated attention on half of the trials. This was included to direct the attention of the participant back to their stage 1 decision without referencing features that were directly related to reward or were probed for transfer. On half of the trials, we asked subjects to select the side of the screen (left or right) on which their chosen first-stage shape was positioned. On the other half of trials, subjects did not see this screen and proceeded directly to the rating screen.

The task was performed in a quiet dark testing room with one researcher present. Participants completed the task in 64 trial blocks with breaks in between. We also tested the WM of each participant with a digit-span test before the experiment began.

#### Analysis

**Figure 2:**
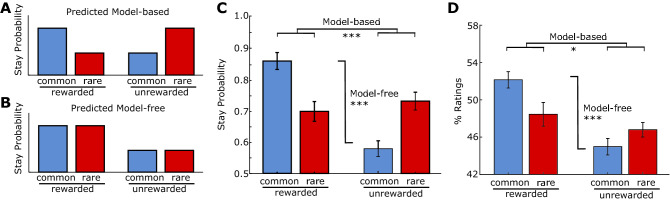
Subjects with explicit instructions showed mixed model-based/model-free choice and transfer. Idealised results of a pure (**A**) model-free and (**B**) model-based approach to the task in rewarded-unrewarded/common-rare trials. (**C**) Contributions of model-free and model-based learning to stay probability, which measures how likely participants are to return to a particular stage 1 colour on the next trial. (**D**) Contributions of model-free and model-based learning to value ratings of the irrelevant stage 1 shapes. % Ratings indicate the average percentile of ratings. Here, we show the ratings of shapes split up by what happened on the most recent trial where that shape was chosen at stage 1. In particular, a shape was rated paradoxically higher if it led to a rare transition and no reward. Error bars represent within-subject standard error of the mean. *p < 0.05, **p < 0.01, ***p < 0.001.

We first analysed choice behaviour using a one-trial-back stay-switch analysis, which is the most widely used method for characterising behaviour on the two-step task. This method quantifies the tendency of a participant to repeat the choice made on the last trial or switch to the other choice, as a function of the outcome and transition on the previous trial. Four indices were used to assess the degree to which choices and shape ratings followed predicted model-based and model-free patterns in line with previous work^23^ (see Eqs. 1–4). If the participant makes use of the task structure (is model-based) and won on the previous trial via a rare transition, they should not re-select the same colour on the next trial in order to maximise reward (Fig. 2A). Conversely, if the participant is unaware of the transitions (is model-free) we would predict that they return to the colour that led to a reward on the next trial (Fig. 2B). By measuring stay probabilities split by conditions on the previous trial (rewarded vs. unrewarded, and common vs. rare) it is possible to establish an estimate of the relative contributions of these strategies to choice.

1 $$Choic{e}_{MB}=stay\,probability\left[\left(common\,rewarded+rare\,unrewarded\right)-\left(common\,unrewarded+rare\,rewarded\right)\right],$$

2 $$Choic{e}_{MF}=stay\,probability\left[\left(common\,rewarded+rare\,rewarded\right)-\left(common\,unrewarded+rare\,unrewarded\right)\right],$$

3 $$Rating{s}_{MB}=ratings\left[\left(common\,rewarded+rare\,unrewarded\right)-\left(common\,unrewarded+rare\,rewarded\right)\right],$$

4 $$Rating{s}_{MF}=ratings\left[\left(common\,rewarded+rare\,rewarded\right)-\left(common\,unrewarded+rare\,unrewarded\right)\right].$$

We analysed the results using MATLAB 2018b and SPSS. Equations 1–4 were used to assess the influences of the model-free and model-based system on choice and ratings. Ratings were organised by rank to ensure even contributions of all participants to the overall results irrespective of their range of ratings and to reduce the influence of outliers. A participant’s behaviour was classed as model-based if *Choice*_*MB*_ > 0. Participants who failed to behave in a model-based fashion were excluded. This resulted in the removal of 2 participants from the analysis. The removal of non-model-based individuals did not affect the statistics of the core findings. One participant was excluded due to their ratings having a standard deviation of less than 20 pixels (2.4 SD below the population mean). Due to the error in width of the human finger it is difficult to ascertain if any variation in their ratings was deliberate. A further participant was excluded due to a computer error, meaning that in total 26 data sets were analysed. Core analysis of stay probability was performed using a mixed logistical regression model including per-subject random effects for all factors. Ratings were analysed using a mixed effects ANOVA with fixed factors of reward, transition and reward × transition plus per-subject random effects of all factors.

### Results 1

First, we examined the contributions of model-free and model-based learning to behaviour. We observed a mixture of both strategies (Fig. 2C), in line with previous studies. Participants were more likely to stick to a given colour in a trial if they received a reward on the previous trial, indicating model-free influence on their choices (linear regression: reward, t(6626) = 16.7, p < 0.0001). Subjects were also more likely to stick to a colour if the reward on a previous trial involved a common transition and showed the converse for an unrewarded trial strategies (linear regression: reward × transition, t(6626) = 12.7, p < 0.0001). These indicate that the model-based system contributed to the decision process. No effect of transition was observed (linear regression: transition, t(6626) = 1.46, p = 0.144).

#### Subjective ratings of task-irrelevant shapes reflect transfer of model-based value

If model-based value was transferred to an irrelevant shape, we would expect that when this shape was chosen and rewarded after a common transition, subsequent ratings of this shape should be *higher* than when a reward was received after a rare transition; the opposite would be true for an unrewarded trial. To analyse for this, we grouped the subjective value ratings for the irrelevant stage 1 shapes according to the conditions (rewarded-unrewarded/common-rare) during the last trial in which that shape had been chosen in stage 1. For example, for a trial where the chevron was rated, we located the last trial where a chevron was chosen, and, if it led to a reward after a common transition, the rating was grouped as “common rewarded”. Remarkably, ratings showed a mixed pattern of model-free (mixed model: main effect of reward, F(1, 100) = 18.0, p < 0.0001) and model-based influences (reward × transition, F(1, 100) = 7.03, p = 0.010) (Fig. 2D), with no effect of transition (F(1, 100) = 0.771, p = 0.384). The model-based (*Ratings*_*MB*_) and model-free (*Ratings*_*MF*_) influence on ratings were (5.5% ± 2.0%, mean ± SEM) and (8.8% ± 2.3%, mean ± SEM) respectively. Overall, these results suggest that when value is assigned to shapes upon reward, both the model-free and model-based system contribute to estimates of value. To rule out the possibility of forward planning at the time of ratings (i.e. that participants were simply estimating the current value of the colour associated with the rated shape when it was last chosen) we conducted two auxiliary analyses (see Supplementary Information). These showed no evidence that outcomes on the ratings trial influenced the ratings of the shape in a model-based fashion, indicating that the model-based influences on ratings we observed reflect value at the time of choice. We also assessed if changes in the modelled value of the colour of the chosen shape in the trials between choice and ratings to look for influences of these intervening trials on ratings. We found that when modelled values increase on these intervening trials, ratings do not increase.

Model-based value thus influenced shape value ratings, despite the fact that stage 1 shapes were outcome-irrelevant, and they were seen at least one trial before. These effects cannot be predicted using the standard model-based learning framework.

#### No impact of attention or working memory on model-based transfer

To quantify the effect of attention, the difference was computed between attention and no-attention trials, separately for each of the four conditions. These attention effects were entered into the same 2 × 2 ANOVA as before. Attention did not affect ratings, or the model-free and model-based influences on ratings (all F(1,100) < 1; Supplementary Fig. S1). We also observed no effect of attention on choice (linear regression: attention, t(6622) = 0.653, p = 0.514), or on model-free (linear regression: attention × reward, t(6622) = 0.081, p = 0.936) and model-based choice behaviour (linear regression: attention × reward × transition, t(6626) = − 1.91, p = 0.0563; Supplementary Fig. S2). In contrast to previous work showing evidence of correlation between WM and model-based behaviour^20^, no evidence was found of a correlation between digit-span and model-based choice or transfer. In line with previous work demonstrating model-free learning of the left/right choice location^32^, we also found a significant tendency of subjects to repeat their position of choice 1 (either left or right) according to a model-free strategy (linear regression: reward, t(6626) = 2.10, p = 0.036) (Supplementary Fig. 3). We also examined whether people tended to re-choose stage 1 shapes after reward. On trials where the chosen stage 1 shape was repeated on the next trial, there was no significant tendency to return to that stage 1 shape after a reward (linear regression: reward, t(6626) = 0.364, p = 0.716).

#### Ratings were best explained by retrospective model-based prediction errors

Our strategy was to fit a standard learning model to the binary choices, then examine how components of this learning drove people to update their subjective ratings. Specifically, the choice model generated various trial-wise reward prediction errors, which we then correlated with the change in ratings of the irrelevant shape chosen on stage 1 of that trial.

We used the original hybrid model by Daw et al.^5^, which permits a mixture of model-based and model-free choices. Model-based and model-free algorithms learn the value of the relevant features that appear in the task by updating their values. There were five free parameters: a learning rate α, a temporal discount factor λ, a mixing weight between model-based and model-free choice ω, the decision precision β, and the choice perseverance π (Supplementary methods). After hierarchically estimating these 5 parameters for each subject (fitted parameters in Supplementary Table S1), the internal value estimates at each trial can be read out, and used to generate various prediction errors.

Crucially, the two stage 1 colours Q_s1_ and four stage 2 shapes Q_s2_ each have a reward value from model-free learning, but the stage 1 colours also have a model-based value that is computed by forward planning with the knowledge of which stage 2 state is likely to follow.

#### Prediction error estimates

**Figure 3:**
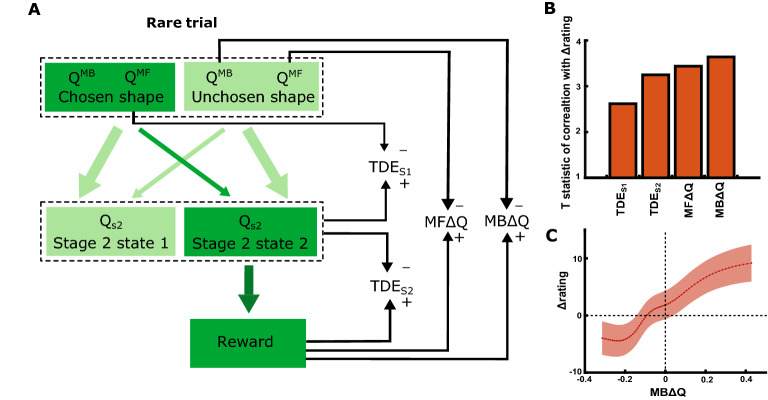
Contribution of reward prediction error estimates to rating changes. (**A**) Four RPEs were examined. On a rare-transition trial, state-values and action-values are updated at the time stage 2 is revealed, and at reward. This leads to a temporal difference error at stage 1 (TDE_S1_) and at stage 2 (TDE_S2_). Retrospectively, the values of the unchosen colour could also be updated, since in future, it is most likely to lead to the current stage 2 shapes. This update in its Q-value reflects the reward either relative to its cached model-free value (MFΔQ), or relative to its expected model-based value (MBΔQ). (**B**) Regression t-statistic between change in ratings and each of the four RPEs. (**C**) Change in participants’ ratings, binned by quantiles of MBΔQ. A sliding window of 20 percentiles was used within participants. Line shows mean of bin, with shaded area reflecting SEM across participants.

To understand which of the computational processes contributed most to the rating values, we computed four possible prediction errors (Fig. 3A):

1. Temporal difference error at stage 1
  $$\mathbf{T}\mathbf{D}\mathbf{E}\mathbf{S}1\hspace{0.17em}=\hspace{0.17em}{Q}_{s2\left[chosen\right]}-{Q}_{s1\left[chosen\right]}^{MF}$$
  TDE_S1_ captures the difference between the estimate of the stage 2 shape’s value and the chosen stage 1 colour’s value. It updates the value of the chosen colour ${Q}_{MF}$ based on the ensuing stage 2 state.
2. Temporal difference error at stage 2
  $$\mathbf{T}\mathbf{D}\mathbf{E}\mathbf{S}2\hspace{0.17em}=\hspace{0.17em}r-{Q}_{s2\left[chosen\right]}$$
  TDE_S2_ captures the difference between the reward and the chosen stage 2 shape’s value (see Eq. 10). It updates the value of the chosen shape ${Q}_{s2}$ based on reward.
3. Retrospective change in model-free action-value
  $$\mathbf{M}\mathbf{F}{\varvec{\Delta}}\mathbf{Q}\hspace{0.17em}=\hspace{0.17em}r-{Q}_{MF\left[retrospective\right]}$$
  MFΔQ captures the difference between the reward and the model-free estimate of stage 1 shape value. It corresponds to a potential change in value of the colour that *would* have led to the most rewarding stage 2 stimulus. This could explain the model-based pattern observed in Fig. 2D. The chosen stage 1 irrelevant shape is up-valued on a common-rewarded trial, whereas the unchosen shape is up-valued on a rare-rewarded trial.
4. Retrospective change in model-based action-value
  $$\mathbf{M}\mathbf{B}{\varvec{\Delta}}\mathbf{Q}\hspace{0.17em}=\hspace{0.17em}r-{Q}_{MB\left[retrospective\right]}$$

MBΔQ captures the difference between the reward obtained and the model-based estimate of stage 1 colour value, again for the colour that *would* have led to the current stage 2 state. This could also lead to the stage 1 shape values being updated in a model-based way, but this time relative to the *planning-based* value of the chosen colour.

Given that these RPEs are strongly correlated, we performed a stepwise regression to assess the relative explanatory power of these RPEs over our behavioural data. The ratings were ranked within subjects as before. On a given trial, we located the previous and next rating of that shape to identify the change in ratings. If a shape was chosen more than once between two consecutive times it was rated, these rating changes were excluded from the analysis. The changes in rating for all trials were fitted to a linear mixed-effects model with a per-subject random intercept, using the MATLAB function fitlme, comparing the four RPEs above as predictors (Fig. 3B). Our choice analysis suggests that the retrospective updates were not directly linked to choice biases, so we did not model the choices using a full retrospective model.

In this cohort, the stepwise linear model revealed that the retrospective MBΔQ on its own provided the best explanation for shape rating behaviour (comparison of MBΔQ model against null model: F(1,1540) = 13.0, p < 0.001). We also reran this analysis with a random intercept and with a random slope and intercept. On both of these analyses, MBΔQ was the best. Overall, the models without random effects had better AIC, so we report those. The likelihood ratio between the two best predictors was only 0.5 log units, providing only weak evidence for MBΔQ over MFΔQ, but *both* of these were retrospective model-based prediction errors. The likelihood ratio between the best retrospective predictor, MBΔQ, was 1.3 log units greater than the best model-free predictor, TDE_S2_. To visualise this relation, the change in ranked rating was binned according to quantiles of RPE for each subject, and the mean and standard error across subjects was plotted (Fig. 3C). When reward was more than expected relative to the colour that would have led to that reward (i.e. MBΔQ > 0), the ratings increased.

### Discussion 1

In this task, we asked whether the perceived value of a task-irrelevant shape is assigned through pure model-free association, or is modulated by the model-based system. In line with the existence of a model-based RPE^5^ and previous findings^6, 11, 12^, our results suggest that information is transferred from the model-based to the model-free system. Our modelling suggests that ratings were best explained by an RPE (MBΔQ) which assigned value based on what *would* have led to a reward given a common transition. As such, participants made use of an internal model when assigning value after a reward to stage 1 shapes.

Whilst we interpret the stage-1 shape ratings as model-free, we cannot rule out that these ratings reflect model-based estimates of the stage 1 shape values. However, there are several reasons why this is highly unlikely. Firstly, by definition the model-based system does not learn shape value, instead it learns the environmental structure^33^. Secondly, participants were explicitly told that colour alone determines transition probability, making the stage 1 shapes outcome-irrelevant. If the model-based system was accurately valuing stage 1 shapes, all stage 1 shapes would be rated equally as they did not affect reward probability. The most parsimonious explanation for our results is that a value learning system that was blind to the task structure (the model-free system) was nevertheless influenced by task structure. Thirdly, the task design ensured that the shape probed was not shown during the stage 1 choice of the current or previous trial. It is implausible that the details of the trial were held in the model-based structure, because the mean distance between choosing a shape and it being probed was 5.31 trials.

One possibility is that the transfer probe may not reflect model-free value but instead the ‘post-arbitration’ value. Arbitration is a putative process that controls the relative contributions of model-free and model-based learning to decision-making^34^. The degree of model-based and model-free choice in the two-stage task varies with structural integrity of white matter tracts from the ventromedial PFC to the medial striatum^28^, suggesting that the medial striatum may play a role in arbitration. Given that previous studies lacked the spatial sensitivity to rule out the presence of the model-based RPE in the medial striatum, it is plausible this RPE may feed into arbitration^5, 7^. However, we did not find an effect of transition or reward on the probability of sticking to a stage 1 shape on the next trial. First-stage decisions were presumably driven by colour, the relevant feature in the task, demonstrating that despite stage 1 shapes being rated in a model-based manner this did not affect decision-making during the task and thus cannot reflect post-arbitration value. The change in shape rating is also not easily explained by a successor representation^18, 35^. Since shapes are not associated with state transitions, when a shape is shown for rating, it is has no reliable connection to the task’s state transition structure.

Attention did not modulate the transfer of value for the chosen shape, which may be because our recall manipulation was insufficient to direct attention to the stimulus. However, an alternative interpretation is that model-based transfer does not require attention. In support of this, digit span tests did not correlate with transfer. This is in contrast to Gershman et al.^6^, who observed that cognitive load decreased the influence of the model-based system on the model-free. One possibility is that our attentional manipulation directed the subjects towards colour and away from shape. Alternatively, the introduction of extra shapes on the attentional manipulation phase of the trial may have inadvertently weakened the memory traces of the correct items by displacing them. Further research should examine if other methods used to increase cognitive load^20^ similarly have no effect on transfer.

The major computational cost of model-based transfer is likely the backwards-search over possible actions within the environmental model, rather than the value and RPE generated using it, which is equally trivial for model-free and model-based RPEs. Since participants’ ability to *use* the internal model for choosing colours was uncorrelated with WM and attention, it is perhaps unsurprising that transfer, which presumably indexes the retrospective generation of a model-based RPE such as MBΔQ, is also not coupled to WM or attention. Given this finding, it remains unclear to what extent transfer requires awareness of model-based values. Therefore, a second experiment was designed to test whether model-based transfer arises without explicit model instructions.

## Experiment 2: No model-based learning without explicit instructions

### Method 2

A second cohort (n = 21) of healthy participants aged 18–35 were tested without explicit knowledge of the relationship between stage 1 shape colour and transitions to stage 2 shapes. They were instructed that “*The stage 1 coloured shape you choose will affect the stage 2 shapes shown*”, but they were not told *which* feature—i.e. colour rather than shape—was causally coupled to the transitions. Stimuli and design were as per experiment 1. Analyses was identical except that here, we did not expect all participants to exhibit model-based choice, so we expanded the analysis to include all participants.

### Results 2

**Figure 4:**
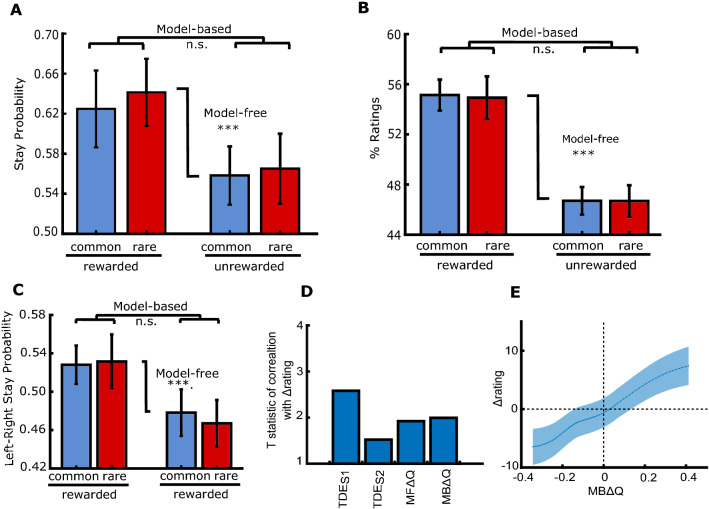
Subjects without explicit instructions showed model-free choice and model-free influences in rating. (**A**) Contributions of model-free and model-based learning to stay probability. Error bars throughout represent within-subject standard error of the mean. (**B**) Contributions of model-free and model-based learning to value ratings of stage-one shapes. % Ratings indicate the average percentile of ratings. (**C**) Participants were more likely to return to the same side of the screen (left or right) in their stage 1 choices if they received a reward. (**D**) t-statistic indicating how well the change in rating was predicted by each of the four RPEs. (**E**) The subjective ratings were changed by intervening trials’ MBΔQ. Trials were binned according to quantiles of the prediction error, line indicates mean, shaded area indicates standard error of the mean across participants. n.s. not significant, *p < 0.05, **p < 0.01, ***p < 0.001.

In these 21 participants, we observed model-free (linear regression: reward, t(5351) = 4.65, p < 0.0001), but not model-based influences on choice (linear regression: reward × transition, t(5351) = 0.333, p = 0.739) (Fig. 4A) and no effect of transition (linear regression: transition, t(5351) = − 0.569, p = 0.569). Only 9 out of 21 participants were model-based (*Choice*_*MB*_ > 0). In contrast to the explicit group, the ratings revealed no evidence of model-based transfer (mixed model: reward × transition, F(1, 80) = 0.007, p = 0.933), however, we observed a strong signature of model-free transfer (main effect of reward, F(1, 80) = 23.9, p < 0.0001) (Fig. 4B). No effect of transition was observed (F(1, 80) = 0.009, p = 0.924). In line with this, the model-based influence on ratings was weak, *Ratings*_*MB*_ (0.198% ± 2.39%, mean ± SEM), but the model-free influence on ratings was very strong, *Ratings*_*MF*_ (16.7% ± 4.26, mean ± SEM). As in experiment 1, there were no effects of attention on ratings (all F > 1). However, we did observe an effect of attention on choice (linear regression: attention, t(5347) = 2.03, p = 0.043), with attention on average increasing stay probability. We observed no effects of attention on model-free (linear regression: attention × reward, t(5347) = 0.414, p = 0.679) and model-based choice behaviour (linear regression: attention × reward × transition, t(5347) = − 0.538, p = 0.591).

Participants could use an alternative strategy where they chose the same first stage stimulus location (i.e. left or right) after receiving a reward. Indeed, participants were more likely to repeat the same location after receiving a reward (linear regression: location repetition ~ reward × transition: main effect of reward, t(5351) = 3.25, p = 0.001) (Fig. 4C), indicating that they used a model-free location-learning strategy that was not observed in the explicit group. There were no effects of transition (t(5351) = 0.127, p = 0.899), or interaction between reward and transition (t(5351) = 0.401, p = 0.689).. We also examined if stage 1 shapes guided choice. Despite observing no shape stay probability effects in the explicit group, we found a significant tendency to repeat their stage 1 shape choice after a reward in the implicit group (linear regression: reward, t(5351) = 4.68, p < 0.0001).

To examine the contributions of prediction errors to the ratings in this group, we applied the same procedure described for the explicit group. The stepwise regression revealed that in the implicit group, TDE_S1_ best explained ratings (comparison of TDE_S1_ model against null model: F(1, 1213) = 8.52, p = 0.00358) (Fig. 4D). TDE_S1_ remained the best predictor when a random intercept, and when a random slope and intercept were included in the analysis. The likelihood ratio between TDE_S1_ and the next best predictor was 2.2 log units. This indicates that in contrast to the explicitly-instructed group, the irrelevant shapes received value updates from the temporal difference error when the second stage stimuli appeared (Fig. 4E).

#### Individual’s use of model-based choice correlates with transfer to ratings

**Figure 5:**
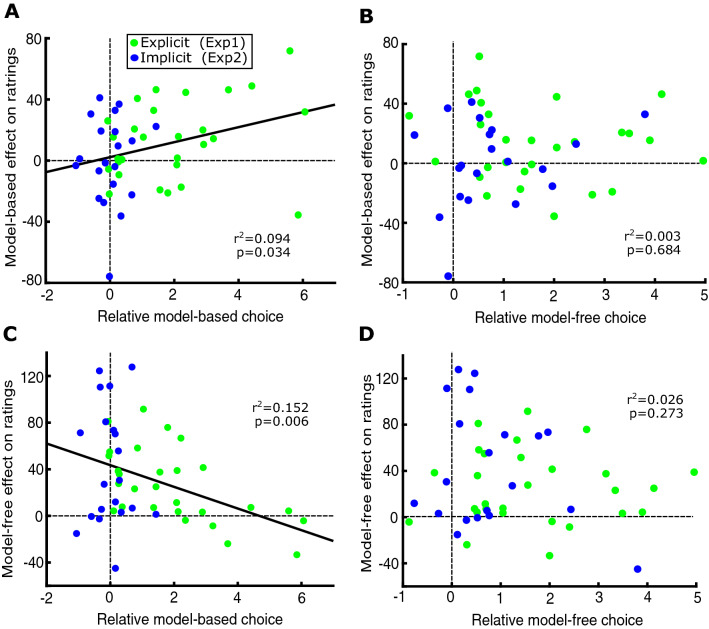
Correlations between choice and ratings. (**A**) Participants who made model-based choices showed the strongest model-based influences on their ratings. (**B**) No significant correlation was observed between model-based ratings and model-free choice. (**C**) Participants who made model-based choices showed smaller effects of model-free valuation on the ratings. (**D**) No significant correlation was observed between model-free ratings and model-free choice.

Finally, we looked across the two experiments at the degree to which individuals exhibited model-based transfer to subjective ratings. The two explicit participants with *Choice*_*MB*_ < 0 were included in this analysis. Model-based choice (log-odds transformed) correlated with the degree of model-based transfer to ratings (r = 0.307, p = 0.034) (Fig. 5A), indicating that both the choice and rating processes might rely on the same model-based prediction errors. Furthermore, a negative correlation between model-based-choice and model-free transfer was also observed (r = 0.390, p = 0.006) (Fig. 5C), meaning that people who planned their choices better were less likely to change their ratings based solely on associated reward. We did not find significant correlations between model-free choice and model-based transfer (Fig. 5B) nor between model-free choice and model-free ratings (Fig. 5D).

### Discussion 2

The implicit task failed to produce accurate model-based choices, and instead, participants formed spurious models of the task structure. During debriefing, it became clear that many participants had invented complex rules such as odd-sided stage 1 shapes being more rewarding. Since this manipulation abolished accurate model-based learning, no model-based transfer would be expected. It may be possible to produce model-based behaviour in implicit conditions; however, the design we adopted encourages participants to look for stage 1 *shape*-related effects on reward, unless they are explicitly told that colour determines the transitions.

Despite observing no effects of our WM manipulation on shape ratings in this group, we did find a significant tendency for individuals to repeat their stage 1 choice on the trial after a WM manipulation. This effect was not present in the explicit group. Given that those with implicit instructions had poor knowledge of the task structure, a likely explanation for this dissociation is that implicit participants were more suggestible to repeating their choice on being made to think back to it. In the absence of a clear strategy, they may have been more likely to be biased by their previous choices.

## General discussion

Our findings suggest that model-based choice is accompanied by a separate process that updates model-free values, as indexed by value ratings, even though this does not manifest in choices. Participants may update action values retrospectively using the model, in the manner of working out what they *ought* to have done, after each outcome. This alternative model-based action value learning approach is consistent with certain accounts of replay^10, 11, 13^ and offline planning^6, 14^, and provides a concrete behavioural index of this previously-invisible form of value updating. Given that choices in the standard two-stage task do not distinguish between these two approaches to model-based behaviour, our findings raise the possibility that this is an important heuristic used by the human brain to produce model-based behaviour. The use of shape ratings to provide a window into this process may be used to explore this possibility further in the future.

**Figure 6:**
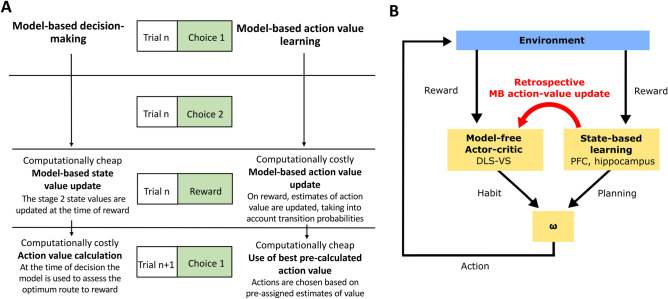
Schematic figures (**A**) Timecourse comparison of traditional model-based decision-making and model-based action value learning. Traditional model-based decision-making involves prospectively assessing action value at the time of decision, whereas model-based action value learning retrospectively assigns value at the time of reward, meaning on decision the action value estimates are already available. (**B**) Schematic of traditional actor-critic architecture for model-free learning with additional model-based transfer marked with red arrows. *ω* arbitration between model-free and model-based system, *RPE* reward prediction error, *VS* ventral striatum, *DLS* dorsolateral striatum, *PFC* prefrontal cortex.

At the heart of model-based action value learning is a retrospective approach to valuation in contrast to the traditionally held view that model-based decision-making is prospective. While both incorporate learning of the transition probabilities and stage 2 stage values, action-value learning involves making retrospective inferences at the time of reward, rather than computing these value estimates of stage 1 options at the time of decision (Fig. 6A). This retrospective approach is consistent with the model used by Toyama et al.^19^ to explain stage 1 choices in the standard two-stage decision task. In their model, the eligibility trace—a measure of how much events contribute to outcomes—can span over possible actions that were not actually performed, and is affected by the task structure. Consequently, RPEs differ on rare versus common trials in a similar fashion to our model. We build on their findings here with a novel paradigm to explore this process, and test more directly for retrospective model-based prediction errors. A different kind of retrospective model-based operation has been reported recently by another group. Moran et al.^36^ found evidence that uncertainty about what led to an outcome can be resolved post hoc by a model-based agent. Their findings confirm the importance of retrospective inferences in which the model-free and model-based systems may work in partnership to drive valuations.

By frontloading the costly work of inference at the time of reward, model-based action value learning may offer a significant advantage over traditional approaches to our understanding of model-based decision-making. By influencing model-free action-value associations, this model-based transfer allows for model-based performance even when traditional model-based system is impaired. Sambrook et al.^7^ suggested that while the performance of a model-free system influenced by model-based information would still exhibit worse performance than a fully model-based approach, if the latter is interrupted due to increased cognitive load this extended model-free system could outperform simple RL. The cost of this approach is inflexibility at the time of decision, in that these action-values are still model-free and therefore unable to take contextual information into account^37^. It remains to be explored to what extent these two methods of model-based decision-making contribute to behaviour.

Recent evidence to support model-based transfer to the model-free system^6, 12^ suggests a significant addition to traditional descriptions of model-free learning is required. Actor-critic computational architectures^38^ for model-free learning are composed of three components: an actor which encodes the action policy, a critic which holds mappings between cues and their predicted value, and a learning signal. Structurally, the VS may hold value estimates of model-free predictions (the critic)^39, 40^ that are trained by dopaminergic RPEs to increase accuracy via D_1_ and D_2_ receptor-mediated plasticity at medium-spiny neurons in the VS^41^. The value estimates made by the VS then inform the dorsolateral striatum (the actor)^39^ to guide actions.

Classical actor-critic architectures fail to incorporate numerous findings including those presented here. The VS has been shown to respond to various model-based properties due to input from the PFC^42^ and hippocampus^43^. VS neurons have been shown to respond in a different way to stimuli that predict equally valued rewards that are sensorially distinct, which is unexpected given model-free system operates solely by reward magnitude^38^. Whilst it is clear the VS subserves multiple functions, the presence of model-based information in the VS supports the ‘multiplexing hypothesis’^2^, which argues that in addition to the model-free RPE, dopamine also encodes model-based reward error generated against internal models. These compound RPEs may then produce complex updates of ‘model-free’ stimulus value. Inhibitory projections from the VS to the ventral tegmental area, or direct cortical input, may support the production of a mixed model-based/model-free RPE which relays back to the VS to update value. Whilst we cannot rule out the possibility that the model-based component of the RPE serves another purpose, a signature essentially identical would likely be required for the results shown by this study. This model-based action value update can be incorporated within the current architecture used to conceptualise model-based and model-free learning (Fig. 6B). Whereas previously the model-free and model-based system have been described as acting independently until arbitration, our findings provide evidence that an internal model of the task structure can influence model-free valuations.

Previous frameworks have also been applied to the training of the model-free system by an internal model that computes action values for chosen and unchosen trajectories through the environmental model. In our task, we suggest this may occur at the time of reward. But our findings are also in keeping with the DYNA architecture, in which the training occurs “offline”, e.g. by replay of experiences from memory^14^. Both accounts are consistent with training being reduced by cognitive load^6^. However, replay of non-experienced outcomes may occur as rapidly as 160 ms-post reward, suggesting a relatively immediate training process^11^. Despite our failure to find effects when manipulating attention between trials, it remains highly speculative at what time and how conscious this process may be. The concept of rumination appears to fit within this domain, and could even be consistent with previously proposed hypotheses of sleep^44^, and it may play an important role in model-based action value learning. In our task a four second limit was applied to the stage 1 choice, which may have curtailed any rumination or replay process. It seems likely that values may be updated both online, as seen in Sambrook et al.^7^, and offline, as observed by Gershman et al.^6^, to implement model-based action value learning.

When multiple features are present in a stimulus, they can independently acquire value over time^45, 46^. In our task, the model-based value of coloured objects in this task ought to be feature specific, as only colour is outcome relevant, but instead, model-based value was distributed to the irrelevant features, in a similar way to reward in feature-value learning^47, 48^.

When knowledge of the task structure was poor (as in the implicit cohort), reward influenced model-free value more strongly than when task structure was known (as in the explicit cohort). In the explicit group we observed no shape stay probability effect, but we did see this in the implicit group. Furthermore, in line with previous findings^32^, we observed a left–right stay probability bias which was greater in the implicit group. These differences therefore appear to relate to knowledge of the task-structure. One interpretation of this dissociation is that it reflects competition between potentially valued task dimensions during learning, during which all task features initially acquire value, before the outcome-relevant features (i.e. stage 1 shape colour) come to dominate over outcome-irrelevant features as observed in the explicit group. Such competition may also explain why we observed a negative correlation between model-based choice and model-free shape ratings. Whilst this is in keeping with evidence that during habit formation, neurons in the dorsolateral striatum shift from encoding several task-relevant aspects to only the most relevant features over time^25^, further work is needed to uncover the factors which arbitrate feature relevance. In the real world many tasks that require knowledge of environmental structure may be split up into a number of subroutines involving simple model-free associations. Transfer of model-based value could provide a mechanism by which a goal-based strategy composed of internal models exerts control over this process^40^. The model-based system may act as a supervising mechanism to down- or upweight task features deemed to be relevant, offering an explanation for the left–right and stage 1 shape stay probability findings.

Our adapted version of the two-stage decision task has here provided evidence that model-based information can influence model-free value in an attention-independent manner. We did not observe this effect in the absence of explicit instructions, indicating that knowledge of the task structure is essential for this process. These findings add to previous work suggesting a re-appraisal of the model-free/model-based dichotomy to include interactions, and also have widespread implications for accounts of emotions^49^ and morality^50^ that make use of this framework. In line with recent opinion^4, 11, 12^, this finding highlights the close relationship between the model-free and model-based systems. Future work is needed to assess the timescale for which transfer persists and to examine the extent to which model-based action value learning can contribute to behaviour.

## Supplementary Information

Supplementary Information.

## Data Availability

Subject data from this study have been deposited in OSF and can be accessed using the following link https://osf.io/e4znv/?view_only=0887fd5ba8714b02aad596beca40314b.

## Code availability

Code used for modelling has been deposited in OSF and can be accessed using the following link https://osf.io/e4znv/?view_only=0887fd5ba8714b02aad596beca40314b.
