# Supplementary material for: Model-based learning retrospectively updates model-free values
Source: Sci Rep. 2022 Feb 11;12:2358. doi: 10.1038/s41598-022-05567-3 (PMC8837618; doi:10.1038/s41598-022-05567-3)
Supplement: Supplementary file 1 — Supplementary Information. [file 41598_2022_5567_MOESM1_ESM.pdf]

Model-Based Learning Retrospectively Updates Model-Free Values

Doody, M., Van Swieten, M.M.H., Manohar, S.G.

Supplementary Figures

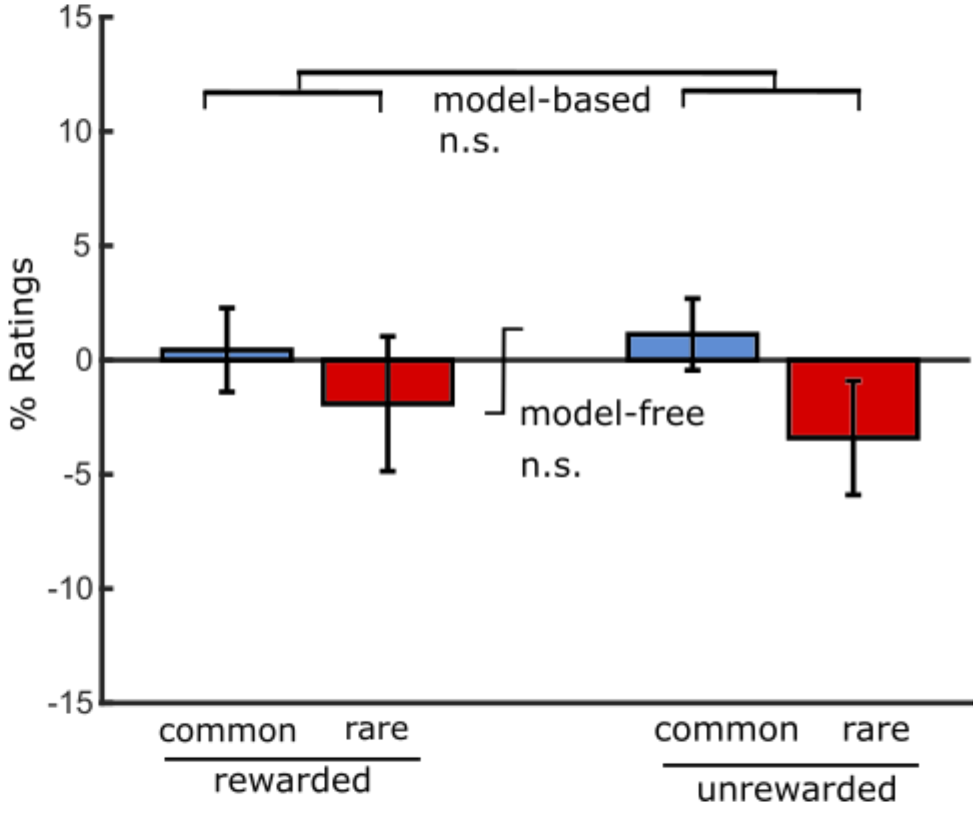

Supp. Fig 1 | Explicit group. % ratings indicate the average percentile of ratings. Attention had no significant effect on model-free or model-based transfer. Therefore, the inclusion of the attentional screen the last time a given shape was shown had no significant effect on the ratings when compared with trials in which the screen was not deployed.

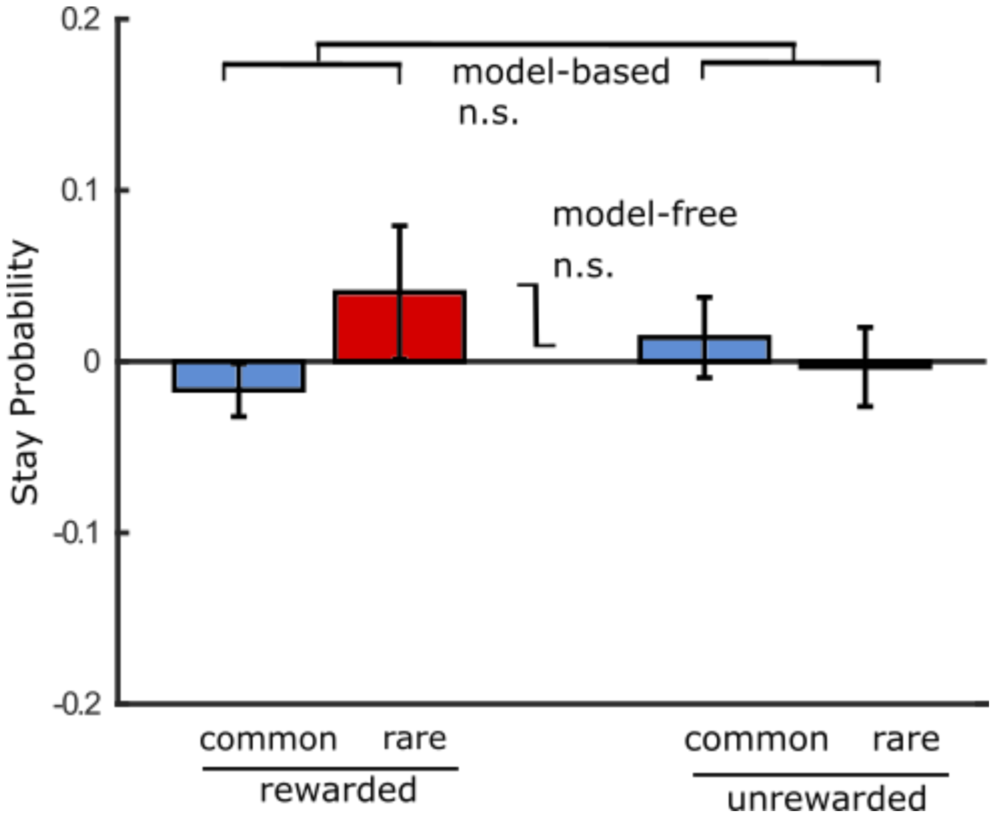

Supp. Fig 2 | Explicit group. The attentional manipulation had no significant effect on stay probability. Therefore, for a given set of win/loss common /rare conditions on a trial, having the attention screen on that trial had no discernible effect on choice on the next trial in comparison to trials without the attention screen.

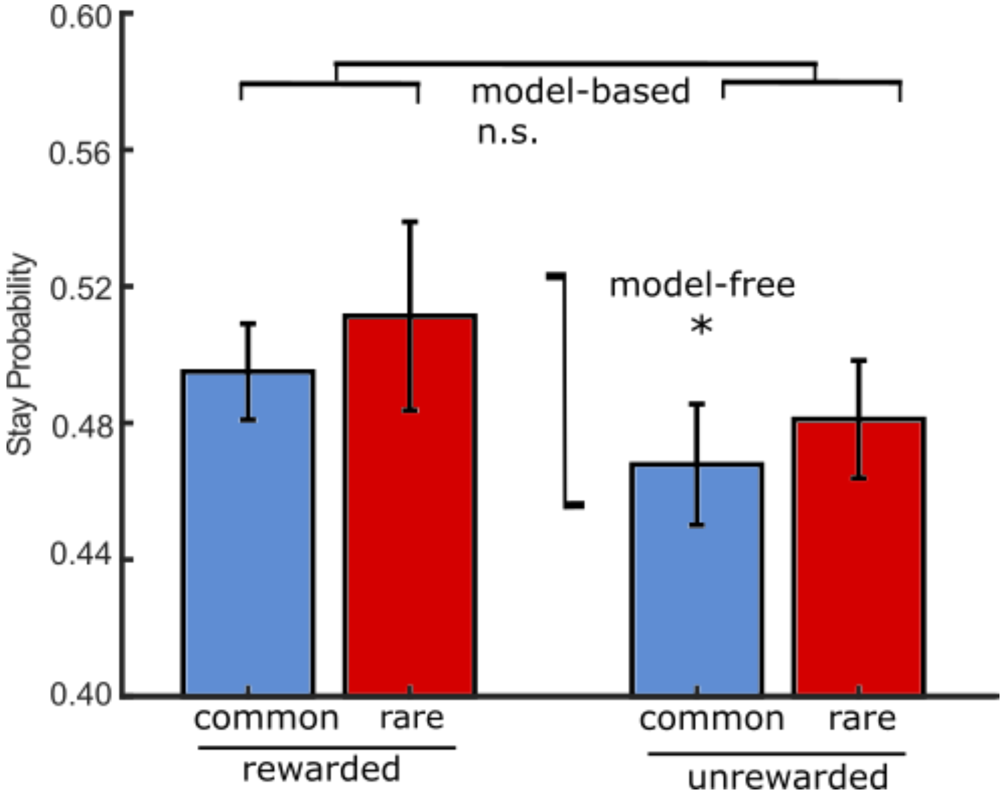

Supp. Fig 3 | Explicit group left-right stay prob. In line with previous reports that participants in the two-stage task may be more likely to return to one side of the screen in stage 1 following a reward, we observed evidence of this in the explicit group. We found no support for a model-based effect on left-right stay probability.

Supplementary Analyses

Hybrid Model-based / Model-free model

We used the original hybrid model by Daw et al.[5] , which permits a mixture of model-based and model-free choices. Model-based and model-free algorithms learn the value of the relevant features that appear in the task in three different pairs. There is one stage 1 pair ($s1 \in \{1,2\}$), representing the two colours. The model is therefore agnostic to the five irrelevant stage 1 shapes, with which these colours were paired. There are two possible stage 2 pairs consisting of two stimuli each ($s2 \in \{3,4,5,6\}$). The subscripts of $s1$ and $s2$ refer to stage 1 and stage 2, respectively.

At the first-stage, model-free 'cached' values were updated using the SARSA (λ) temporal difference algorithm. This algorithm learns to maximise the total outcome by strengthening or weakening

associations between the first-stage state and the first-stage actions, depending on whether the action was followed by a reward, and on what second-stage state ensued:

$$\Delta Q_{s1}^{\mathrm{MF}} = \alpha\delta_{s1} + \alpha\lambda\delta_{s2}, \tag{5}$$

where δ refers to the trial-by-trial prediction error used to update the stimulus weighted by the learning rate α. The prediction error for the first and second stage, $\delta_{s1}$and δ_{s1}respectively, are computed as the difference between the expected value and the obtained reward (r):

$$\delta_{s1} = r_{s1}(t) + Q_{s2[chosen]}(t) - Q_{s1}^{\mathrm{MF}}(t), \tag{6}$$

$$\delta_{s2} = r_{s2}(t) + Q_{s3}(t) - Q_{s2[chosen]}(t). \tag{7}$$

Note that $r_{s1}(t) = 0$ because no reward is delivered after the first-stage choice, and $Q_{s3}(t) = 0$ because the task only has two stages. Q-values for the four second-stage stimuli were updated according to the reward prediction errors[51]:

$$\Delta Q_{s2[chosen]} = \alpha\delta_{s2}. \tag{8}$$

The parameter λ is a temporal discounting parameter, which connects the two stages and allows the second-stage reward prediction error to influence first-stage choices. If $\lambda = 1$, the reward on stage 2 drives the update of the stage 1 value immediately, whereas if $\lambda = 0$, stage 1 values are updated only vicariously. The parameter λ therefore accounts for the main effect of reward as observed in the analysis of first-stage stay-switch behaviour, but not for an interaction of reward and state.

Model-based values were calculated for each first-stage stimulus and every trial in a forward-looking manner by multiplying the state value of the best second-stage option with the state transition probabilities. These probabilities were specified explicitly in the model, rather acquired by learning, because previous simulations showed that learning of state transitions quickly converge to stable values[5]:

$$Q_{s1,1}^{\mathrm{MB}} = 0.7 \times max\left(Q_{s2,3}, Q_{s2,4}\right) + 0.3 \times max\left(Q_{s2,5}, Q_{s2,6}\right), \tag{9}$$

$$Q_{s1,2}^{\mathrm{MB}} = 0.3 \times max\left(Q_{s2,3}, Q_{s2,4}\right) + 0.7 \times max\left(Q_{s2,5}, Q_{s2,6}\right). \tag{10}$$

The hybrid model then computes the stage 1 choice using a weighted combination of the model- based (Q_{s1}^{MB}) and model-free (Q_{s1}^{MF}) values. The first-stage Q-values were computed in the following way:

$$Q_{s1}^{\mathrm{hybrid}} = \omega Q_{s1}^{\mathrm{MB}} + (1-\omega) Q_{s1}^{\mathrm{MF}}, \tag{11}$$

where $0<\omega<1$ is a weighting parameter for the model-based strategy and $(1-\omega)$ is the weighting factor for the model-free strategy.

A first-stage choice depends on the relative difference in stimulus values between $Q_{s1,1}$ and $Q_{s1,2}$ and the choice C on the previous trial, which takes on the value 1 when the current choice equals the previous choice. The parameter π captures perseveration on the colour of the first-stage choice. Using the softmax choice function, the probability of choosing a first-stage stimulus was computed according to:

$$P_1 = 1/\left(1 + exp\left(-\beta\left(Q_{s1,1}^{\mathrm{hybrid}} - Q_{s1,2}^{\mathrm{hybrid}}\right) - \pi C\right)\right), \tag{12}$$

and for the second-stage:

$$P_3 = 1/\left(1 + exp\left(-\beta(Q_{s2,3} - Q_{s2,4})\right)\right), \tag{13}$$

where $1/\beta$ is the softmax temperature that controls the stochasticity of choices, which we assume to be the same for both stages.

Model fitting procedure

We used a hierarchical model-fitting strategy that takes into account the likelihood of individual participant choices given the individual participant parameters and also the likelihood of the individual participant parameters given the parameter distribution in the population. The hierarchical fitting follows an iterative expectation-maximization algorithm, which regularises individual participants' parameter fits, rendering them more robust to over-fitting. The parameters were estimated for the explicit learning group and implicit learning group separately.

As per Wunderlich et al.[1], we transformed [0,1]-bounded parameters (λ ,α, ω) into a Gaussian scale using the logistic function:

$$\alpha = 1/\big(1 + exp(-a)\big), \quad (14)$$

and the [0, ∞)-bounded parameters, (β, π), were logarithmically scaled using the exponential function:

$$\beta = exp(b). \quad (15)$$

The model parameters are denoted by Greek letters and their respective Gaussian transformations by Latin letters. Normally distributed parameters allow for the use of parametric tests to identify differences between sessions.

Gradient-based optimisation algorithms are not guaranteed to converge to a global maximum and could instead converge to local maxima. To avoid this, the algorithm was initialised with a range of starting parameters and the iteration with the highest likelihood value was chosen to make further inference.

Table S1| Best-fitting model parameter estimates. Separately shown for the explicit and implicit group.

**Explicit group**	**β**	**α**	**λ**	**ω**	**π**
25th percentile	2.41	0.44	0.67	0.29	0.11
Median	3.66	0.51	0.77	0.39	0.18
75th percentile	4.37	0.69	0.83	0.57	0.27
**Implicit group**	**β**	**α**	**λ**	**ω**	**π**
25th percentile	1.48	0.08	0.23	0.16	0.03
Median	2.37	0.30	0.36	0.20	0.06
75th percentile	3.29	0.52	0.43	0.27	0.13

Auxiliary colour value analyses

One possible interpretation of the shape rating effects we observed is that participants may rate shapes based on the colour value of the grey shape at the time of rating. This would necessitate remembering

the colour of rated shape when previously chosen, and rating the shape according to a model-based evaluation of the act of choosing that colour. While such an effect could account for our findings, any subject employing such a tactic would also be influenced by rewards revied between last choosing a shape and rating that shape.

To exclude this possibility, we performed two additional analyses. If the alternative approach were employed, we would expect the effect of reward on the ratings trial to update the colour value and consequently influence ratings. Reward should increase ratings if the chosen colour on the ratings trial is the same colour as the rated shape when it was last chosen, and decrease ratings if the colours were incongruent. To assess for this, we split the ratings according to whether the last time that shape was presented, it was in the colour chosen on the current trial, or in the other colour. Then we ask whether current trial reward and transition affect ratings on the current trial. There was no effect of current trial reward, or transition, or interaction, when the colour matched. There was also no interaction of colour-match-vs-nonmatch with current reward.

We also looked at the modelled value of the colour of the shape when it was last chosen. If this value has increased since the shape was last chosen, e.g. due to intervening trials with rewards for that colour, then the rating should be correspondingly higher. In other words, the planning explanation predicts that events after the shape was last chosen should influence its subsequent rating. We identified when the shape was last chosen, found the model-based value (Q^{MB}) of that colour at that time, and compared it with the current MB value of the same colour (at time of rating). We split trials according to whether the Q^{MB} has increased vs decreased since it was last chosen. Ratings did not increase when the values increased.

51. Rummery, G. A. & Niranjan, M. On-line q-learning using connectionist systems (1994). http://mi.eng.cam.ac.uk/reports/svr-ftp/auto-pdf/rummery_tr166.pdf
